# Propagation-based Phase-Contrast X-ray Imaging at a Compact Light Source

**DOI:** 10.1038/s41598-017-04739-w

**Published:** 2017-07-07

**Authors:** Regine Gradl, Martin Dierolf, Lorenz Hehn, Benedikt Günther, Ali Önder Yildirim, Bernhard Gleich, Klaus Achterhold, Franz Pfeiffer, Kaye Susannah Morgan

**Affiliations:** 1 Chair of Biomedical Physics, Department of Physics, Technical University of Munich, James-Franck-Str. 1, 85748 Garching, Germany; 2 Munich School of BioEngineering, Technical University of Munich, Boltzmannstr. 11, 85748 Garching, Germany; 3 Institute for Advanced Studies, Technical University of Munich, Lichtenbergstrasse 2 a, 85748 Garching, Germany; 4 Department of Diagnostic and Interventional Radiology, Klinikum rechts der Isar, Technical University of Munich, Ismaninger Str. 22, 81675 München, Germany; 5 School of Physics and Astronomy, Monash University, Clayton, Victoria, 3800 Australia; 6 Comprehensive Pneumologie Center (CPC), Institute of Lung Biology and Disease, Helmholtz Zentrum München, Member of the German Lung Center for Lung Research (DZL), Ingolstädter Landstr. 1, 85764 Neuherberg, Germany; 7 Max-Plank-Institute for Quantum Optics, Hans-Kopfermannstr. 1, 85748 Garching, Germany

## Abstract

We demonstrate the applicability of propagation-based X-ray phase-contrast imaging at a laser-assisted compact light source with known phantoms and the lungs and airways of a mouse. The Munich Compact Light Source provides a quasi-monochromatic beam with partial spatial coherence, and high flux relative to other non-synchrotron sources (up to 10^10^ ph/s). In our study we observe significant edge-enhancement and quantitative phase-retrieval is successfully performed on the known phantom. Furthermore the images of a small animal show the potential for live bio-imaging research studies that capture biological function using short exposures.

## Introduction

Since their discovery, X-rays have strongly influenced a variety of scientific domains. In particular their ability to penetrate matter has been used to visualize the inner structures of opaque objects. Through the construction of synchrotron radiation facilities from the late 1980s the available X-ray flux and coherence has experienced a large improvement, enabling high speed imaging and leading to new phase-contrast imaging techniques that take advantage of the increased spatial and temporal coherence. However these large-scale facilities have limited beam time, are high in cost and are not available for routine applications. Therefore, there is a strong interest in moving experiments from synchrotrons to laboratory sources^1^ for greater availability (e.g. enabling longitudinal studies over weeks and days) and as a step towards clinical imaging^2^.

Until now the performance gap between synchrotrons and laboratory sources has been large. The Munich Compact Light Source (MuCLS) sits in this gap by providing a partially spatial coherent, near monochromatic X-ray beam set at an energy between 15 keV and 35 keV and with a flux of about 10^10^ ph/s, depending on the selected X-ray energy and machine tuning (brilliance of 4.8 × 10^9^ ph s^−1^ mm^−2^ mrad^−2^ (0.1% BW)^−1^ for an X-ray energy of 25 keV)^3, 4^. Furthermore, the low divergence of 4 mrad of the beam allows the X-ray detector to move several meters away from the source to increase the spatial coherence without compromising the available flux. This enables laboratory experiments which were previously limited to large scale synchrotron facilities.

**Figure 1:**
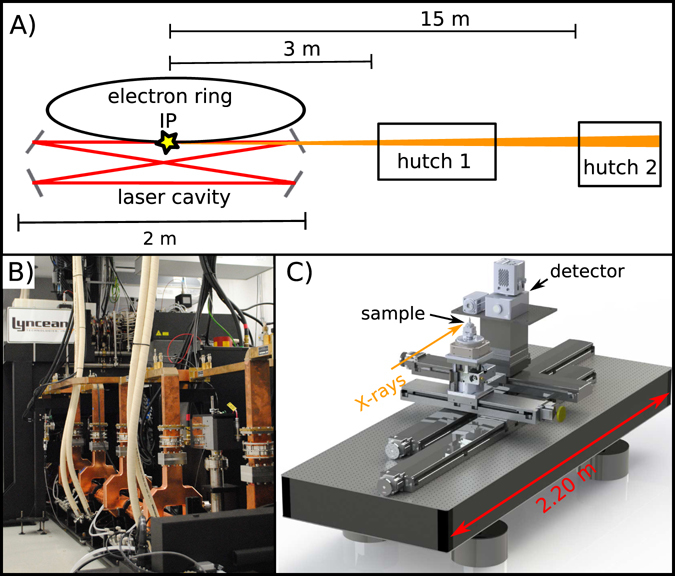
(**A**) Schematic of the Munich Compact Light Source (MuCLS) facility. The facility consists of three main parts. The first one is the production of the X-rays, the second and third are the experimental hutches 1 and 2 placed downstream of the interaction point (IP) of the electron and laser beam. (**B**) Electron accelerator of the MuCLS machine. (**C**) CAD-view of the setup located in hutch 1.

A schematic overview of the MuCLS facility is given in Fig. 1(A). The MuCLS is a compact synchrotron, which generates X-rays via inverse Compton scattering. The photons are produced at the interaction point (IP) where an intense laser beam (1 μm wavelength) collides with a counter-propagating electron beam (E = 20–45 MeV). This X-ray beam is guided to two end-stations. One is placed around 3 m (hutch 1) from the interaction point and the second one is 15 m away (hutch 2). In this report, the focus lies on the near experimental hutch. Figure 1(C) shows the setup installed for propagation-based phase-contrast imaging and high-resolution micro-tomography. There the distance between the sample and the detector can be varied between zero and two meters. The beam divergence (4 mrad) leads to a field of view (FOV) of about 16 mm in diameter 4 meters away from the interaction point. This medium-sized FOV allows for imaging samples of several millimeters to centimeters in width. In particular, a whole mouse lung fits within this field of view.

A fundamental step for a significant contrast enhancement in X-ray imaging has been achieved by the transfer of the phase-contrast imaging principle from visible light to X-rays in 1965 by Bonse and Hart^5^. Particularly with the advent of synchrotrons several phase sensitive methods have arisen, including X-ray grating interferometry^6–8^, analyser-based refraction contrast^9^ and propagation-based X-ray phase-contrast imaging^10, 11^. These phase-contrast mechanisms are particularly useful when imaging objects that deliver insufficient contrast in absorption imaging. They have been applied in biomedical research (e.g. imaging soft tissue, airways), material science (e.g. imaging low Z materials) and show potential as a new clinical diagnostic tool^2^.

In order to investigate the potential of the MuCLS for time-sequence imaging of respiratory processes, we focus here on propagation-based imaging (PBI). PBI is particularly useful in this context because only a single exposure is required to produce an image, whereas other techniques, for example grating-based imaging, often require several exposures to reconstruct a phase-contrast image. It would also be possible to conduct single-exposure techniques on this setup that utilise a single-grid^12, 13^ or speckle-tracking^14, 15^, but we leave those topics for future studies. While PBI experiments do not require a strictly monochromatic beam, and may thus also be performed at conventional laboratory sources^16^, an X-ray beam with a certain amount of spatial coherence is necessary, hence such laboratory sources must have a small effective source size. In addition, many research applications require high flux to avoid motion blur and/or capture changes in biomedical structure or function. In biological specimens, one may want to capture motion (e.g. of the lungs^17, 18^ or of inhaled particles moving along airways^19^) or a time-resolved response to a treatment (e.g. in the depth of liquid lining the airways^20^). A small focal spot in a conventional X-ray source will often mean the flux is limited by the heat load on the target, although this can be circumvented to an extent by using, for example, a liquid metal jet target^21, 22^, which also produces high brilliance X-rays^23^. Magnification of the sample when using a divergent beam (e.g. from an X-ray target) typically means larger detector pixels are used than at the synchrotron, and in the case of a X-ray/optical scintillator system, a thicker scintillator. This results in a more efficient detector system, enabling high speed imaging. However, magnification can also affect the ability to capture phase fringes in a flux-efficient dynamic imaging setup, as explored in the discussion of this paper. Dynamic PBI experiments have so far taken place at synchrotrons, but the development of high-flux compact X-ray sources like the MuCLS and the liquid metal jet source suggest that these experiments may be now possible in the laboratory.

In this report we show that the flux and coherence of the quasi-monochromatic X-ray beam provided by the MuCLS allows phase-contrast imaging by simply increasing the sample-to-detector distance. We demonstrate that short exposure times (∼50 ms) are possible at a cm-sized FOV in a laboratory environment with a resolution of around 50 μm. In the following section, the first results of propagation-based phase-contrast X-ray imaging obtained at the MuCLS are presented on well-known phantoms (nylon fibers and perspex spheres) and a biomedical sample (a mouse).

## Results

**Figure 2:**
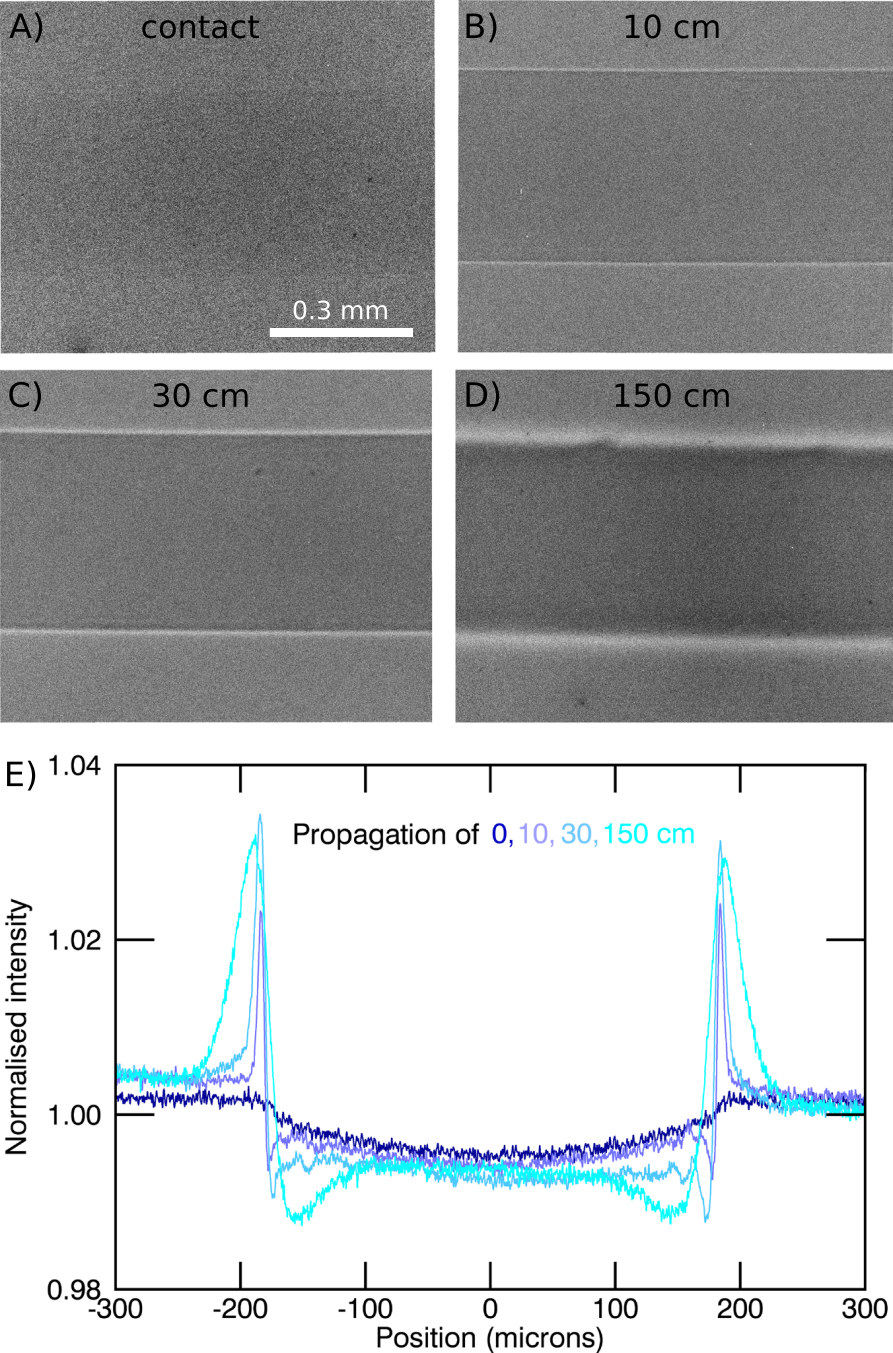
Nylon fiber (350 μm diameter) imaged with a detector pixel size of 0.65 μm for different propagation distances. (**A**) In the contact regime the contrast of the fiber is very weak. In the propagation range between (**B**) and (**C**) the edge-enhancement delivers contrast to better visualise the fiber. (**D**) Due to the source-blurring the edges are smeared out for large distances, but the increased width at this distance means the edge-enhancement can be detected with larger pixels. (**E**) Intensity profiles across the images in (**A–D**) (exposure time = 240 s).

**Figure 3:**
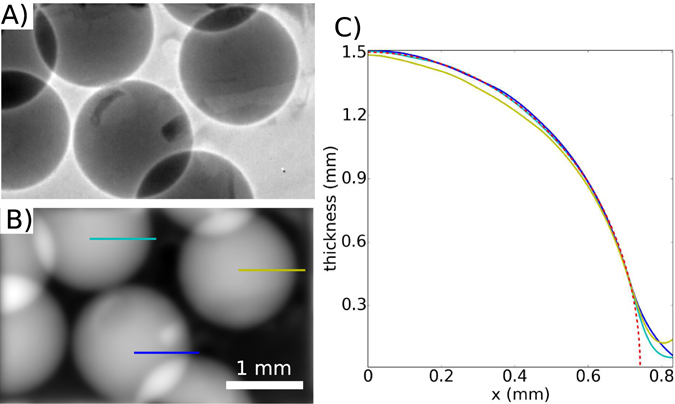
PMMA spheres (**A**) edge enhanced image, (**B**) thickness reconstruction and (**C**) intensity profile along the lines highlighted in (**B**) compared with the theoretical thickness of perfect PMMA spheres (red dashed line). Overlying imperfections are largely due to the kapton tape holding the spheres in place.

**Figure 4:**
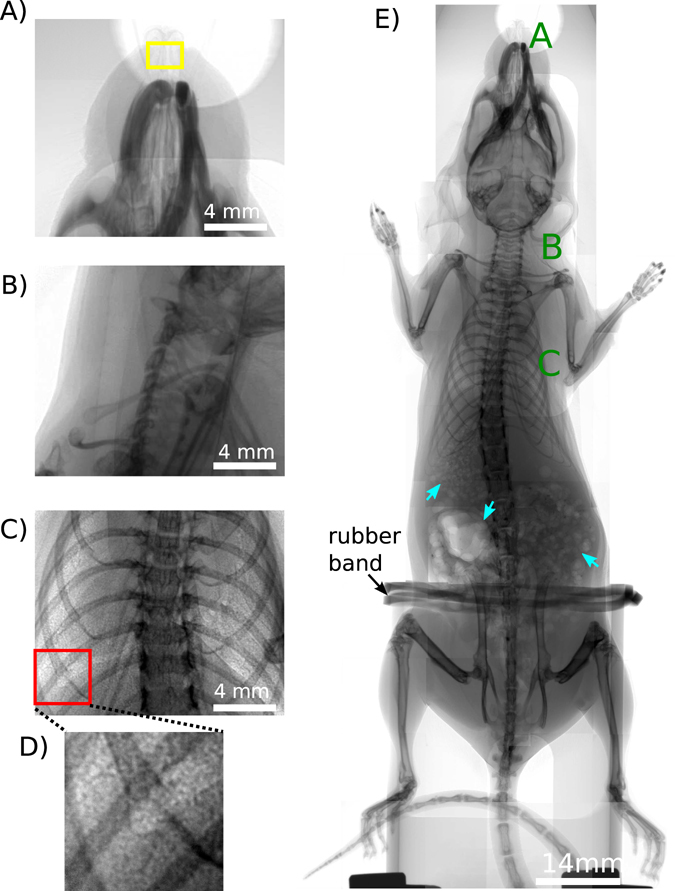
Panels (A)–(C) show the interesting areas in lung and airway imaging in an *ex-vivo* mouse model. (**A**) The nasal airways. The yellow box highlights the area which is imaged in Fig. 5 with the high resolution setup. (**B**) Trachea region (for A and B detector pixel size = 6.5 μm, exposure time = 1 s), (**C**) Lung (detector pixel size = 13 μm, exposure time = 1 s), (**D**) is a magnification of the red box in (**C**) and shows the speckle pattern which results from the air sacs in the lungs, the alveoli. (**E**) Overview scan of the *ex-vivo* mouse imaged with propagation-based X-ray phase-contrast, composite image using 1 m propagation and 10 second exposure times (detector pixel size = 6.5 μm). The green letters A, B, C indicates the regions where the images shown in panels A–C were taken. The mouse was fixed with a rubber band. In the stomach gas bubbles are visible (highlighted with blue arrows).

**Figure 5:**
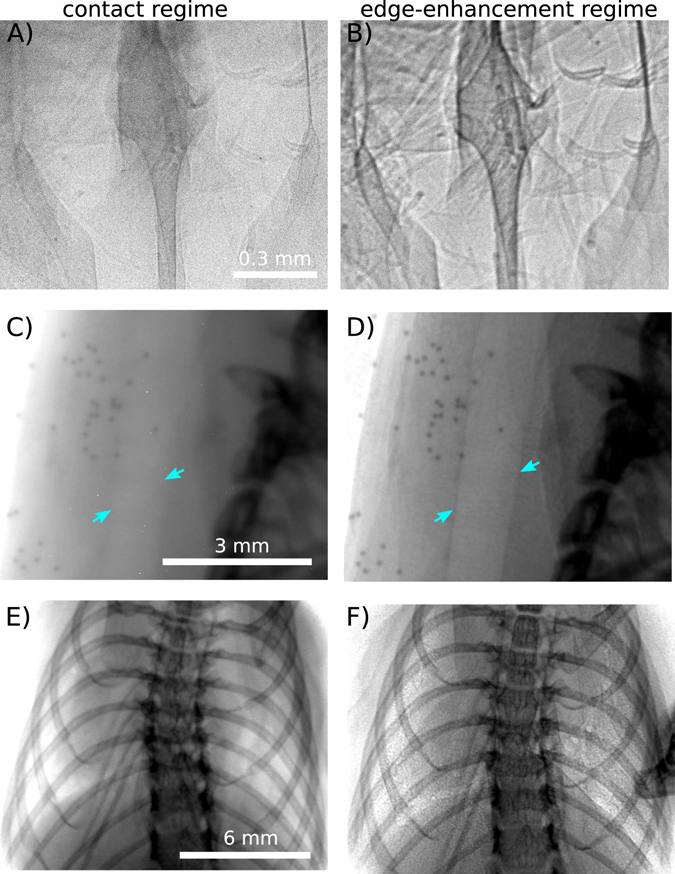
The contrast in all three regions of interest is increased by increasing the detector-sample distance to the edge-enhancement regime. (**A**) and (**B**) shows the nasal airways imaged with a detector pixel size of 0.65 μm and exposure time of 180 s. In (**B**) the sample-detector distance is increased to 30 cm. In panels (C) and (D) the trachea region with a detector pixel size of 6.5 μm and exposure time of 1 s is shown. The sample-detector distance is 1.5 m in (**D**). The blue arrows highlight the edges of the trachea. The lungs are displayed in (**E**) and (**F**) with a detector pixel size of 13 μm and exposure time of 1 s. The sample-detector distance is increased to 1.6 m in (**F**) and is less than 2 cm for (**A**), (**C**) and (**E**).

**Figure 6:**
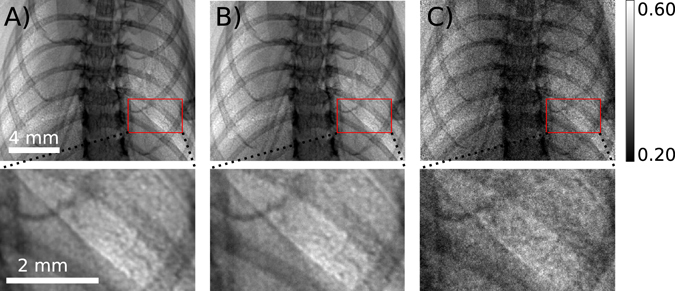
*Ex vivo* murine lungs captured with (**A**) 10 s (flat-field 150000 ADUs, dark-current 2000 ADUs), (**B**) 1 s (flat-field 15000 ADUs, dark-current 200 ADUs) and (**C**) 0.05 s exposure times (flat-field 900 ADUs, dark-current 100 ADUs) (sample-detector distance = 1.5 m, detector pixel size = 13 μm). The red boxes highlight the magnified areas in the second row.

By extending the free space propagation between the sample and the detector up to 2 m, the X-ray wavefield diffracts and self-interferes to produce characteristic edge-enhanced images. This effect is clearly visible with the Nylon fiber shown in Fig. 2 and the perspex spheres shown in Fig. 3, good examples of light density materials with X-ray properties comparable to soft tissue. We also observe phase effects from the biomedical sample in Figs 4 and 5, the lungs and respiratory tract of a mouse. The images are obtained with two different detector systems, which allow us to choose between different resolutions in the micron range and different fields of view. The systems are described in detail in the methods section. The beam divergence of 4 mrad leads to a medium sized beam diameter of around 14 mm in diameter, 3.5 m away from the interaction point. This FOV in combination with the high flux allows the whole mouse lung to be captured with a single exposure of a few tens of milliseconds (Fig. 6).

### Nylon thread

Nylon fibers with a diameter of 350 μm were imaged with the high resolution setup. Figure 2 shows the results for propagation distances between contact and 150 cm. At an energy of 25 keV the fibers can be considered as basically pure phase objects, as their absorption contrast is very weak (e.g. Fig. 2A). However, the increasing phase contrast is clearly visible in Fig. 2(B–D), primarily at the edges where the phase gradient is large. In Fig. 2(E) the line profiles for the different distances are shown. The fringes increase in width and intensity with increasing sample-detector distance. We can adjust the sample-detector distance by balancing the increasing fringe width and visibility seen with the smoothing effects of the source width. For this 0.65 μm pixel system, propagation distances in the cm range (below 50 cm) deliver the best edge contrast. Due to the source blurring at larger distances the fringes are smeared out, but these wider fringes are useful in the case of a larger pixel size.

### PMMA spheres

Here we show that the propagation-based phase-contrast images of phantoms on this setup can provide quantitative sample thickness values. PMMA spheres with a diameter of 1.5 mm are imaged with a detector pixel size of 6.5 μm, a sample-detector distance of 1 m and exposure time of 120 s. Figure 3(A) shows the edge-enhanced image. In Fig. 3(B) the projected thickness of the spheres is reconstructed with the single-distance phase-retrieval algorithm developed by Paganin *et al*.^24^. Using this algorithm the thickness of a sample of a single known material can be reconstructed. For the PMMA spheres (C5O2H8) the complex refractive index values *δ* = 4.228e-07 and *β* = 1.796e-10 are used for the 25 keV image. The intensity profiles along the lines shown in Fig. 3(B) are plotted in Fig. 3(C), alongside the theoretical thickness for a sphere with a diameter of 1.5 mm (red). The thickness values from the phase-retrieval fit well with the theoretical values, differing only at the edges, where the reconstruction produces the ‘softer’ edges that are typical of this algorithm^25^.

### Lung and Airways in a small animal model

Figure 4(E) shows an *ex-vivo* mouse imaged using several projections. The green letters mark the particularly interesting areas in lung and airway imaging, which are the nasal airways (Fig. 4A), the trachea region (Fig. 4B) and the lungs (Fig. 4C). These features are normally barely discernible in conventional absorption imaging, but their visibility can be enhanced using phase-contrast methods. Figure 5 shows the effect of increasing the sample-detector distance, with particularly good edge-enhancement from air/tissue interfaces. The contact images were obtained with sample-detector distances of less than 2 cm. The visibility of the lungs and airways increases by increasing the propagation distance up to 1.6 m, with three different detector setups used here (see methods). Figure 5(A and B) were obtained with the smallest detector pixel size used in this report, 0.65 μm, therefore the contrast of the nasal airways increases remarkably by changing the propagation distance to 30 cm. The edges of the nasal airways are clearly enhanced, as well as the hairs on the nose of the mouse. The visibility of the beads in Fig. 5(C and D) shows that this setup allows us to image the clearance of inhaled debris (modelled by these beads) along the airway away from the lungs, a clearance mechanism which is impaired by cystic fibrosis^19^.

Imaging moving samples, like the lungs, also requires short exposure times. Figure 6 shows the lung captured with a propagation distance of 1.5 m and exposure times of (A) 10 s, (B) 1 s and (C) 0.05 s. All images are only flat-field and dark-current corrected. There is no remarkable increase in the quality of the image by increasing the exposure time from 1 s to 10 s. By decreasing the exposure time to 0.05 s, the image gets nosier, but still the quality of the image is sufficient to perform lung motion measurements.

## Discussion

This report shows that a compact synchrotron X-ray source can be used to increase the contrast of low density materials and tissues by simply increasing the sample-detector distance. For a single-material we are able to reconstruct the quantitative thickness of the sample with Paganin’s single-distance algorithm. Furthermore the edge-enhancement we obtain is sufficient to render the lungs and airways visible in X-ray imaging. We can adjust the sample-detector distance to achieve a useful fringe width and visibility, taking into account source blurring that occurs at large sample-to-detector distances relative to the source-to-sample distance^26^. For the detector system with the larger pixel size of 6.5 μm and 13 μm we obtained the best contrast for a distance of about 1.5 m. For the smaller pixel size of 0.65 μm, the best contrast was found around 30 cm for the samples we tested. This is consistent with theoretical expectations in that the width of the first phase contrast fringe^26^ is comparable to the relevant point spread function of each detector at these distances and the source blurring is matched to the detector blurring^27^.

We also see an advantage to the low divergence of the MuCLS. In propagation-based phase-contrast imaging, divergent sources will require a greater sample-to-detector propagation distance to achieve an image displaying the same relative phase effects as captured at a synchrotron. If an experiment is to be transferred from a synchrotron (close to plane wave illumination) to a laboratory source (point source illumination), and the images are to be equivalent (ie. any Fresnel fringes are the same fraction of the sample size in the image), the sample-to-detector propagation distance required at the divergent source is *z*_*div*_ = *Mz*, according to the Fresnel scaling theorem^37^. Here, *M* = (*z*_*div*_ + *R*)/*R* is the magnification when the sample is distance *R* from the source and *z* is the sample-to-detector propagation distance when using plane wave illumination (well-approximated at the synchrotron, where *M* is typically within a few percent of 1.0). Since the magnification and propagation distance are co-dependent when considering a point source, inserting the definition of *M* into the propagation length scaling shows that this requires *z*_*div*_ = *R*_*z*_/(*R *− *z*). The entire divergent setup will have a source-to-detector size of

1 $${d}_{src-to-det}=R+{z}_{div}=R+Rz/(R-z),$$

which is positive for *R* > *z*, asymptotes to *R* + *z* for large *R* (e.g. a synchrotron) and has a minimum of ${d}_{src-to-det}\mathrm{=4}z$ at *R *= 2*z* and *M* = 2. This means that the total length of a divergent source setup (${d}_{src-to-det}$) must be at least four times the desired synchrotron propagation distance (*z*) to achieve an equivalent image. For small animal lung imaging, a propagation distance of z > 1 m is typical at the synchrotron^35^, requiring that an equivalent laboratory setup is at least 4 metres from source to detector. Therefore, if phase effects are sought that are equivalent to those at synchrotron propagation distances of >1 m (typical of many samples >1 cm), it is desirable that the laboratory source is low divergence (like the MuCLS) in order to illuminate both the sample and detector by as much of the available flux as possible in a >4 m long setup. This allows the best use of the available flux for fast propagation-based imaging. Note that if reduced phase effects are sufficient, the setup could be more compact. A low divergence source like the MuCLS enables a source-to-sample distance of several metres, which also contributes to the coherence of the beam, all without significant loss of flux.

Another advantage of the MuCLS is the relatively large field of view which allows imaging of a mouse lung with a single shot. This study shows that we are able to use the MuCLS for dynamic respiratory imaging. Previous laboratory-based respiratory studies have shown the ability to detect lung structure differences in live mice using X-ray images with exposure times extending over several breaths^28, 29^. Increased diagnostic power should be available via improved spatial resolution if the lungs can be imaged without motion blur. Since mice breathe naturally at around 100 times/min, exposure times of less than 200 ms are desirable^30^. To reduce motion blurring it is also possible to capture images over several breaths and average images from the same point in every breath cycle, as the motion is repeated with every breath^31^, therefore permitting shorter exposures without reducing SNR (provided there is not a time-dependent treatment response occurring over this timespan). This kind of high speed imaging also enables spatially-resolved measurements of functional lung health, for example via X-ray particle imaging velocimetry (PIV)^17^ based tracking of lung motion. To capture particles moving along the airway surface, frame rates of less than 1 s are necessary as particles move via normal clearance mechanisms at 2–3 mm/minute. This method can be used to study the effects of a treatment on airway clearance mechanisms^19^. With the MuCLS we are able to capture projections of the mouse lung with exposure times of about 50 ms (at 25 keV). This gives access to dynamical respiratory imaging in the variants discussed above. If we can capture these kinds of biological events, this can help research in better understanding physiology (e.g. aeration at first breath of life^32^) and in better treating disease (e.g. observing the effects of a treatment^19^).

We conclude that the results of this study show that propagation-based phase-contrast imaging is feasible with the Munich Compact Light Source and that the source fulfills the requirements for future studies of airways and lungs of *in vivo* small animal models which have previously been primarily limited to large synchrotron radiation facilities. These studies will enable important pre-clinical lung and airway research.

## Methods

### Imaging Setup

Imaging was performed at the Munich Compact Light Source. Detailed descriptions of the working principles of the MuCLS are published by the manufacturer Lyncean Technologies, Inc. and in several previous publications^3, 4, 33, 34^. Specimens were located 3.5 meters from the source. The distance between source and sample can be adjusted between 3.2 m and 4.7 m by a motorized linear stage. For all experiments, quasi-monochromatic X-rays at 25 keV were chosen with flux up to 2.4 · 10^9^ ph/s and a source size of 39 μm × 45 μm. Propagation distances (sample-to-detector distances) between zero and 1.6 m were used to visualize phase effects. The distances were adjusted by a motor-driven translation stage. Three different detector systems were used to capture the images: (1) A 20 μm thick Gadox scintillator (Gd2O2S:Tb) deposited on a fiber optic plate coupled to an Andor Zyla 5.5 sCMOS camera, with a resulting detector pixel size of 6.5 μm; (2) A 20 μm thick Gadox screen (Gd2O2S:Tb) deposited on a 2:1 fiber optic taper coupled to the Andor Zyla 5.5 sCMOS camera, resulting in a detector pixel size of 13 μm; (3) Conversion of X-rays into visible light by a 10 μm thick LSO scintillator in front of an optical system (Hamamatsu AA50) with a 10x optics (NIKON) coupled to a Andor Neo 5.5 sCMOS camera which leads to a detector pixel size of 0.65 μm. For the lens coupled system longer exposure times are necessary as the efficiency is lower than for a scintillator fiber-optic coupled detector and the FOV is just 1.66 mm × 1.4 mm, therefore only a small part of the available flux can be used. The FWHM of the point spread functions of the detectors were measured around 50 μm (Zyla camera setup with 1:1 taper), 90 μm (Zyla camera setup with 2:1 taper) and 7 μm (Neo camera setup). Various exposure times (0.05 s–240 s) and propagation distances were chosen in this experiment for useful propagation-based X-ray phase-contrast imaging at the MuCLS. The surface entrance dose was estimated at 4 mGy for a 1 s X-ray exposure of the mouse lung, consistent with synchrotron lung imaging^35^.

### Image Reconstruction

The projections obtained in this study were all flat-field and dark-current corrected. Propagation-based phase-contrast imaging uses the free-space propagation of a coherent X-ray beam to create contrast. After passing through the sample the wavefront is distorted as a consequence of the phase-shift imposed by the sample. The propagation of the distorted X-ray wavefront gives rise to Fresnel diffraction fringes in the image which enhance edges and interfaces present in the sample^10, 36^. This edge enhancement is especially useful for weakly-attenuating samples, which are otherwise not seen in the image. Additionally propagation-based images provide quantitative thickness maps with a single-distance phase-retrieval algorithm. Here the non-iterative phase-retrieval algorithm developed by Paganin *et al*. has been used^24^. For a single-material sample, the thickness can be reconstructed.

All images are cropped from the full detector area to the region of interest.

### Samples

To demonstrate the edge enhancement effect, weakly-attenuating samples were chosen. The samples for the proof-of-principle experiments were nylon threads and perspex spheres with known diameter and well-known material composition.

Eight week-old pathogen-free female C57BL/6N mice (Charles River Laboratories) were used for this analysis. Mouse had free access to water and rodent laboratory chow. Mouse experiment were carried out in strict accordance with the recommendations in the Guide for the Care and Use of Laboratory Animals of the National Institutes of Health. The protocol was approved by the ethical committee of the regional governmental commission of animal protection (Munich). Images were taken between 1 hour and 48 hours after the death of the mouse and some hair was removed from the neck area.
